# Factors Determining Sensitivity and Resistance of Tumor Cells to Arsenic Trioxide

**DOI:** 10.1371/journal.pone.0035584

**Published:** 2012-05-10

**Authors:** Serkan Sertel, Margaret Tome, Margaret M. Briehl, Judith Bauer, Kai Hock, Peter K. Plinkert, Thomas Efferth

**Affiliations:** 1 Department of Otorhinolaryngology, Head and Neck Surgery, University of Heidelberg, Heidelberg, Germany; 2 Department of Pathology, University of Arizona, Tucson, Arizona, United States of America; 3 Pharmaceutical Biology (C015), German Cancer Research Center, Heidelberg, Germany; 4 Department of Pharmaceutical Biology, Institute of Pharmaceutical Biology, University of Mainz, Mainz, Germany; University of Texas Health Science Center at San Antonio, United States of America

## Abstract

Previously, arsenic trioxide showed impressive regression rates of acute promyelocytic leukemia. Here, we investigated molecular determinants of sensitivity and resistance of cell lines of different tumor types towards arsenic trioxide. Arsenic trioxide was the most cytotoxic compound among 8 arsenicals investigated in the NCI cell line panel. We correlated transcriptome-wide microarray-based mRNA expression to the IC_50_ values for arsenic trioxide by bioinformatic approaches (COMPARE and hierarchical cluster analyses, Ingenuity signaling pathway analysis). Among the identified pathways were signaling routes for p53, integrin-linked kinase, and actin cytoskeleton. Genes from these pathways significantly predicted cellular response to arsenic trioxide. Then, we analyzed whether classical drug resistance factors may also play a role for arsenic trioxide. Cell lines transfected with cDNAs for catalase, thioredoxin, or the anti-apoptotic bcl-2 gene were more resistant to arsenic trioxide than mock vector transfected cells. Multidrug-resistant cells overexpressing the *MDR1*, *MRP1* or *BCRP* genes were not cross-resistant to arsenic trioxide. Our approach revealed that response of tumor cells towards arsenic trioxide is multi-factorial.

## Introduction

Arsenic is a natural semimetal in soil, water and air. It exists as red arsenic (As_2_S_2_), yellow arsenic (As_2_S_3_), white arsenic (As_2_O_3_, arsenic trioxide), phenylarsine oxide (C_6_H_5_AsO), and as salts of sodium, potassium and calcium [1]. Since ancient times arsenic was used for medical purposes [2]. Arsenic was appreciated as Fowler's Solution for many diseases in the 18^th^ and 19^th^ century, *i.e.*, syphilis, cancer, ulcers, etc. [3]. In the 20^th^ century, Paul Ehrlich, the founder of modern chemotherapy, found the arsenical salvarsan, which was the standard therapy against syphilis for decades [4]. On the other side, arsenic compounds can be poisonous [5]. The revival of arsenic in modern medicine was initiated by Chinese scientists showing dramatic regression rates of acute promyelocytic leukemia by arsenic trioxide [6]. These findings were subsequently corroborated in clinical studies in the USA [7].

Various molecular determinants of the biological effect of arsenic trioxide have been elucidated. It promotes the degradation of the oncogenic fusion protein of the PML and retinoic acid receptor α (RARα) genes which arises from t(15;17) translocation in acute promyelocytic leukemia, resulting in induction of cellular differentiation [7], [8]. Apoptosis is selectively induced in malignant cells through enhancement of reactive oxygen species and activation of caspases [9]–[12]. Cells can arrest in the G1 or G2/M phases of the cell cycle after treatment with arsenic trioxide [12]. Tumor angiogenesis is targeted by arsenic trioxide through inhibition of vascular epithelial growth factor production [13].

While focusing on mono-specific drugs without adverse effects on normal tissues, it turned out that drug resistance frequently occurs. Subpopulations of cancer cells with specific point mutations in target proteins can survive attacks of mono-specific drugs due to reduced binding affinity to these drugs. They overgrow the entire tumor population resulting in drug-resistant phenotypes, as in the case of Gleevec® resistance [14]. Therefore, it has recently been proposed that multi-target attacking drugs maybe superior by avoiding development of resistance to single mono-specific drugs. The development of multi-kinase inhibitors represents an example for this novel treatment concept.

The aim of the current study was to investigate sensitivity and resistance of tumor cells towards arsenic trioxide. For this reason, we first analyzed transcriptome-wide microarray-based mRNA expression by bioinformatic approaches (COMPARE and hierarchical cluster analyses, Ingenuity signaling pathway analysis) to identify novel molecular determinants for response of the cell line panel of the National Cancer Institute (NCI), USA, towards arsenic trioxide [15].

A second aim was to analyze whether classical determinants of resistance towards established anti-cancer drugs may also play a role in arsenic trioxide resistance. To this end, anti-oxidative stress response genes as well as multidrug resistance transporters have been tested for their influence on arsenic trioxide resistance. A major obstacle of cancer therapy is the development of cross-resistance and even worse multidrug resistance [16]–[17]. The role of the drug transporters P-glycoprotein (Pgp, MDR1, ABCB1) and multidrug resistance related protein 1 (MRP1, ABCC1) has been discussed with contradictory results [18]–[21] and it is unclear whether or not arsenic trioxide is transported by these two multidrug resistance pumps. Therefore, we have readdressed this question. Furthermore, we analyzed the breast cancer resistance protein (BCRP, ABCG2) whose relevance for resistance to arsenic is unknown as yet.

Furthermore, it has been claimed that arsenic trioxide generates reactive oxygen species (ROS) [22]–[24] leading to apoptosis. The role of ROS-detoxifying enzymes for arsenic trioxide has been investigated. Again, conflicting data have been reported [25]–[27]. Since most of these studies only measured enzymatic activities, we used cell lines transfected with cDNAs for catalase or thioredoxin to clarify whether or not these genes confer resistance to arsenic trioxide.

## Materials and Methods

### Cell Lines

The panel of 60 human tumor cell lines of the Developmental Therapeutics Program of the NCI, USA, consisted of leukemia (CCRF-CEM, HL-60, K-562, MOLT-4, RPMI-8226, SR), melanoma (LOX-IMVI, MALME-3M, M14, SK-MEL2, SK-MEL28, SK-MEL-5, UACC-257, UACC-62), non-small cell lung cancer (A549, EKVX, HOP-62, HOP-92, NCI-H226, NCI-H23, NCI-H322M, NCI-460, NCI-H522), colon cancer (COLO205, HCC-2998, HCT-116, HCT-15, HT29, KM12, SW-620), renal cancer (786-0, A498, ACHN, CAKI-1, RXF-393, SN12C, TK-10, UO-31), ovarian cancer (IGROV1, OVCAR-3, OVCAR-4, OVCAR-5, OVCAR-8, SK-OV-3) cell lines, cell lines of tumors of the central nervous system (SF-268, SF-295, SF-539, SNB-19, SNB-75, U251), prostate carcinoma (PC-2, DU-145), and breast cancer (MCF-7, NCI/ADR-Res, MDA-MB-231, Hs578T, MDA-MB-435, MDA-N, BT-549, T-47D). Their origin and processing have been previously described [28].

Multidrug-Resistant Tumor Cell Lines: Leukemic CCRF-CEM cells were maintained in RPMI 1640 medium (Invitrogen, Eggenstein, Germany) supplemented with 10% fetal calf serum in a humidified 5% CO_2_ atmosphere at 37°C. Cells were passaged twice weekly. All experiments were performed with cells in the logarithmic growth phase. P-glycoprotein/multidrug resistance gene 1 (*MDR1*)-expressing CEM/ADR5000 cells were maintained in 5000 ng/ml doxorubicin. The establishment of the resistant subline has been described [29].

The multidrug-resistance gene 1 (MRP1)-expressing HL-60/AR subline was continuously treated with 100 nM daunorubicin. The establishment of this cell line has been reported (Brügger et al., 1999) [30]. Sensitive and resistant cells were kindly provided by Dr. J. Beck (Department of Pediatrics, University of Greifswald, Greifswald, Germany). Breast cancer cells transduced with control vector (MDA-MB-231-pcDNA3) or with cDNA for the breast cancer resistance protein BCRP (MDA-MB-231-BCRP clone 23) were maintained under standard conditions as described above for CCRF-CEM cells. The generation of the cell lines followed a published protocol [31]. The cell lines were continuously maintained in 800 ng/ml gentamicin (Invitrogen, Karlsruhe, Germany). Oxidative stress-related cell lines: The mouse thymic lymphoma-derived WEHI7.2 parental cell line was obtained from Dr. Roger Miesfeld (University of Arizona, Tucson, AZ). Cells were maintained in Dulbecco’s Modified Eagle Medium - low glucose (Invitrogen, Carlsbad, CA) supplemented with 10 % calf serum (Hyclone Laboratories, Logan, UT) at 37°C in a 5 % CO_2_ humidified environment. Stock cultures were maintained in exponential growth at a density between 0.02 and 2×10^6^ cells/ml. WEHI7.2 cells stably transfected with and overexpressing human bcl-2 (Hb12), constructed and maintained as described in [32], were also obtained from Dr. Miesfeld. Thioredoxin overexpressing cells (THX) were constructed by stably transfecting human thioredoxin into WEHI7.2 cells, then selecting and maintaining clones as described [33]. THX cells express 1.8-fold more thioredoxin than the parental cells [33]. Catalase overexpressing cells were constructed by stably transfecting WEHI7.2 cells with a vector containing rat catalase as described [34]. The CAT38 clone expressing 1.4-fold parental cell catalase activity was selected and maintained in 800 µg/ml G418 (GIBCO-BRL). Hydrogen peroxide resistant cells (200R) were developed by subculturing parental cells in the presence of fresh H_2_O_2_ every three days as described [35]. This procedure resulted in a population of cells that is 2.8-fold more resistant to 200 µM H_2_O_2_ than the parental cells. 200R cells were maintained in the presence of 200 µM H_2_O_2_. Any variant normally grown in the presence of drug was cultured in the absence of drug for one week prior to each experiment.

### Drug Response

**Figure 1:**
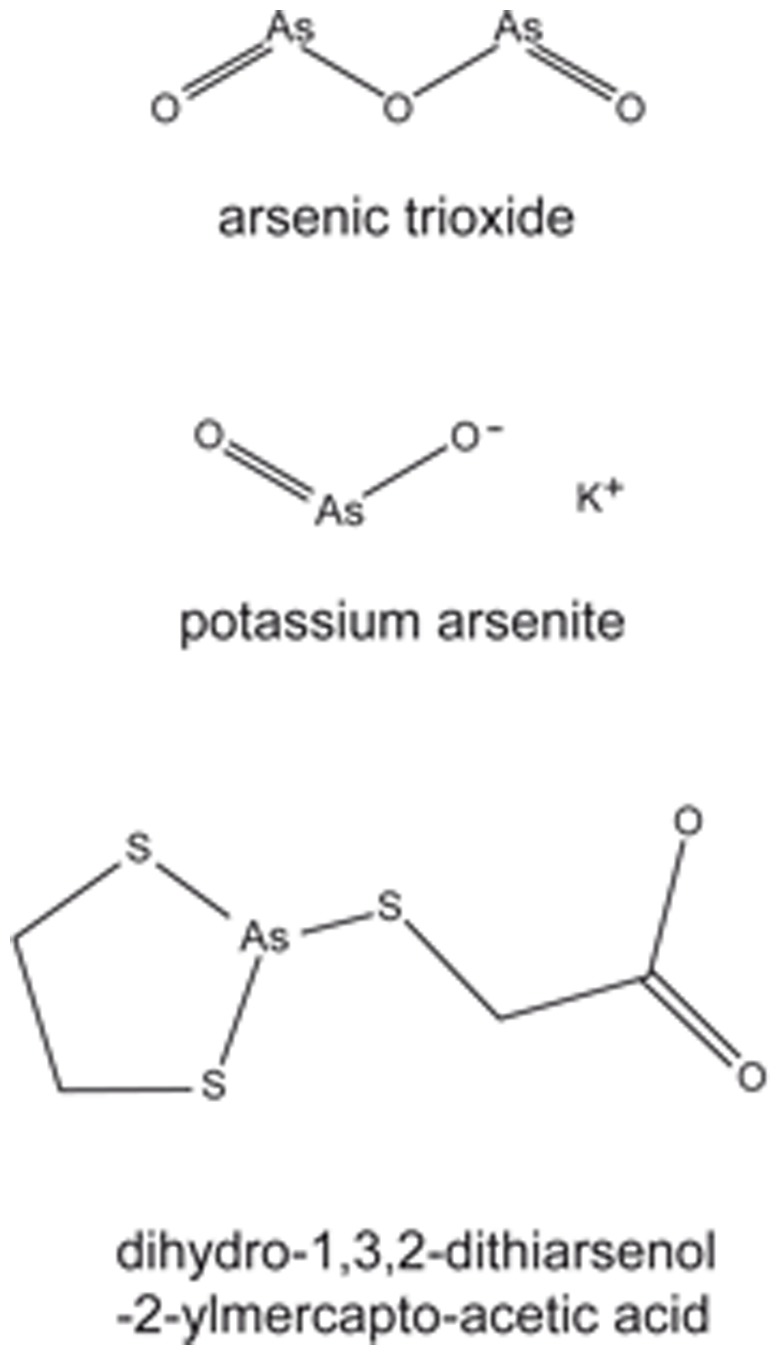
Chemical structures of arsenic trioxide, potassium arsenite, and dihydro-1,3,2-dithiarsenol-2ylmercapto-acetic acid.

The sulforhodamine B assay for the determination of drug sensitivity in the NCI cell lines has been reported [36]. The inhibition concentration 50% (IC_50_) values for free and formulated arsenic trioxide (Trisenox) as well as for other arsenic compounds (potassium arsenite, dihydro-1,3,2-dithioarsenol-2-ylmercapto-acetic acid) and standard cytostatic drugs have been deposited in the database of the Developmental Therapeutics Program of the NCI (http://dtp.nci.nih.gov). Their chemical structures are shown in **Figure 1**.

Growth Inhibition Assay: The *in vitro* response to drugs was evaluated by means of a growth inhibition assay as described [37]. Aliquots of 5×10^4^ cells/ml were seeded in 24-well plates and drugs were added immediately at different concentrations. Arsenic trioxide was used in different doses to allow calculation of IC_50_ values. Cells were counted 7 days after treatment with the drugs. The resulting growth data represent the net outcome of cell proliferation and cell death.

MTS assay: The response of WEHI7.2 parental cells and WEHI7.2 cell variants towards arsenic trioxide was measured using the MTS assay (Promega Corp., Madison, WI, USA) as described previously [38]. Briefly, cells were plated at 1.5×10^4^ cells/well in 100 µl medium in a 96-well plate and incubated in the absence or presence of the indicated concentrations of arsenic trioxide for 48 hrs. Relative absorbance was measured by incubating the cells for 3 hrs at 37°C with the MTS solution, prepared and used according to the manufacturer’s protocol (Promega Corp., Madison, WI), and reading at 490 nm using a Microplate Autoreader (Bio-Tek Instruments, Winooski, VT). Response was calculated as percent absorbance of untreated control. The IC_50_ represent the mean of three independent experiments. The degrees of resistance were calculated by dividing the IC_50_ of transfected cell lines and multidrug-resistant cell lines, respectively, by the IC_50_ value of their corresponding mock vector control or parental cell line.

### Microarray-Based Bioinformatic and Statistical Analyses

Cell lines of the NCI-60 panel were grown under standard conditions [29]. RNA isolation and microarray hybridization procedures have been described [39]–[40]. The microarray data have been deposited at the website of the NCI Developmental Therapeutics Program (http://dtp.nci.nih.gov). Hierarchical cluster analysis is an explorative statistical method and aims to group at first sight heterogeneous objects into clusters of homogeneous objects. Objects are classified by calculation of distances according to the closeness of between-individual distances. All objects are assembled into a cluster tree (dendrogram). The merging of objects with similar features leads to the formation of a cluster, where the length of the branch indicates the degree of relatedness. The procedure continues to aggregate clusters until there is only one. The distance of a subordinate cluster to a superior cluster represents a criterion for the closeness of clusters as well as for the affiliation of single objects to clusters. Thus, objects with tightly related features appear together, while the separation in the cluster tree increases with progressive dissimilarity. Previously, cluster models have been validated for gene expression profiling and for approaching molecular pharmacology of cancer [39], [41]. Cluster analyses applying the WARD method were done by means of the WinSTAT program (Kalmia Co., Cambridge, USA). Missing values are automatically omitted by the program and the closeness of two joined objects is calculated by the number of data points they contained. In order to calculate distances of all variables included in the analysis, the program automatically standardizes the variables by transforming the data with a mean = 0 and a variance = 1. To visualize the relationships between the IC_50_ values for arsenic trioxide and mRNA expression levels by cluster analyses, cluster image maps were formed.

For COMPARE analysis, the mRNA expression values of genes of interest and IC_50_ values for free and formulated arsenic trioxide (Trisenox) of the NCI cell lines were selected from the NCI database (http://dtp.nci.nih.gov). The mRNA expression has been determined by microarray analyses as reported [39]. COMPARE analyses were performed to produce rank-ordered lists of genes expressed in the NCI cell lines. The methodology has been described previously in detail [42]. Briefly, every gene of the NCI microarray database was ranked for similarity of its mRNA expression to the IC_50_ values for the corresponding compound. To derive COMPARE rankings, a scale index of correlations coefficients (R-values) was created. In the standard COMPARE approach, greater mRNA expression in cell lines correlate with enhanced drug resistance, whereas in reverse COMPARE analyses greater mRNA expression in cell lines indicated drug sensitivity.

The Ingenuity Pathway Analysis software (IPA) (Ingenuity Systems, Mountain View, CA, USA; http://www.ingenuity.com) was utilized to identify networks and pathways of interacting genes and other functional groups in genomic data. Using the IPA Functional Analysis tool we were able to associate biological functions and diseases to the experimental results. Moreover, we used a biomarker filter tool and the Network Explorer for visualizing molecular relationships.

Pearson’s correlation test was used to calculate significance values and rank correlation coefficients as a relative measure for the linear dependency of two variables. This test was implemented into the WinSTAT Program (Kalmia Co.). Pearson’s correlation test determined the correlation of rank positions of values. Ordinal or metric scaling of data is suited for the test and transformed into rank positions. There is no condition regarding normal distribution of the data set for the performance of this test. We used Pearson’s correlation test to correlate microarray-based mRNA expression of candidate genes with the IC_50_ values for arsenic trioxide.

The Chi^2^-test was applied to bivariate frequency distributions of pairs of nominal scaled variables. It was used to calculate significance values (*P*-values) and rank correlation coefficients (R-values) as a relative measure for the linear dependency of two variables. This test was implemented into the WinSTAT program (Kalmia Co.). The Chi^2^-test determines the difference between each observed and theoretical frequency for each possible outcome, squaring them, dividing each by the theoretical frequency, and taking the sum of the results. Performing the Chi^2^-test necessitated defining cell lines as being sensitive or resistant to arsenic trioxide. This was done by taking the median IC_50_ value (log_10_ = −5.346 M for formulated arsenic trioxide and log_10_ = −5.467 M for free arsenic trioxide) as a cut-off threshold.

## Results

### Cross-resistance of Arsenic Compounds in the NCI Cell Line Panel

**Figure 2:**
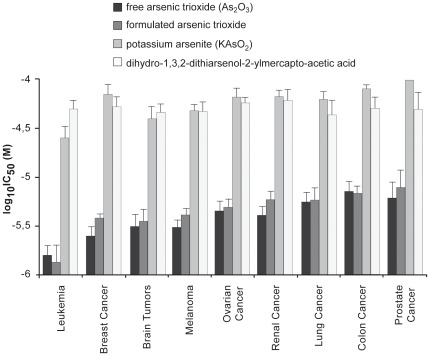
IC_50_ values of four arsenicals for the NCI cell line panel. Mean values and SEM of IC_50_ are grouped according to the tumor origin of the cell lines.

The NCI database contained 9 arsenic-containing compounds, of which five were inactive or only minimally active against the cancer cell lines tested. The four cytotoxic arsenicals were free and formulated arsenic trioxide as well as potassium arsenite, dihydro-1,3,2-dithioarsenol-2-ylmercapto-acetic acid. The inactive or weakly active arsenicals were arsenic(III) 2,3-dimercapto succinic acid, simethyl arsinic acid, lithiume arsenate (Li_3_AsO_4_), sodium arsenic tungsten polyoxymetalate hydrate, and arsenic acid (H_3_AsO_4_) trilithium salt. These substances have been investigated over a dose range from 10^−8^ to 10^−4^ M in 60 tumor cell lines and IC_50_ values have been calculated thereof. The IC_50_ values for the four cytotoxic arsenic compounds are shown in **Figure 2**. Free and formulated arsenic trioxides were more cytotoxic than the two other arsenicals. Leukemia cell lines were more sensitive than cell lines from other tumor types. Among cell lines of solid cancers, cell lines from brain tumors, melanoma, or breast cancer were most sensitive to free or formulated arsenic trioxide, whereas colon or prostate cancer cell lines were most resistant. Cell lines from lung or kidney cancer showed intermediate sensitivity.

We correlated the IC_50_ values for free and formulated arsenic trioxide with those of other arsenic-containing compounds (potassium arsenite, dihydro-1,3,2-dithioarsenol-2-ylmercapto-acetic acid). As shown in **Table 1**, the IC_50_ values for free and formulated arsenic trioxide were highly correlated (*P* = 4.14×10^−14^). Furthermore, the IC_50_ values for arsenic trioxide of the cell line panel were significantly correlated with the IC_50_ values for potassium arsenite and dihydro-1,3,2-dithiarsenol-2-ylmercapto-acetic acid, indicating that the cell lines reveal cross-resistance to arsenic-containing drugs.

**Table 1:** Cross-resistance between arsenic trioxide and other arsenic compounds in the NCI cell line panel.

	free As_2_O_3_	formulated As_2_O_3_
formulated As_2_O_3_	4.14×10^−14*^	N/A
potassium arsenite (KAsO_2_)	1.76×10^−12*^	2.51×10^−9*^
dihydro-1,3,2-dithiarsenol-2-ylmercapto-acetic acid	0.04565*	n.s.

Log_10_ IC_50_ values obtained from SRB assays have been subjected to Pearson’s correlation test.

N/A, not applicable; N.S. not significant (*P*>0.05). * denotes significant correlation.

### Gene-hunting Approach

#### COMPARE and Cluster Analyses of Microarray-Based mRNA Hybridization

We applied a pharmacogenomic approach to explore novel molecular determinants of sensitivity and resistance to arsenic trioxide. We mined the genome-wide mRNA expression database of the NCI and correlated the expression data with the IC_50_ values for arsenic trioxide. This represents a hypothesis-generating bioinformatic approach, which allows the identification of novel putative molecular determinants of cellular response towards arsenic trioxide.

Standard COMPARE analysis was performed to identify genes, while expression was associated with arsenic trioxide resistance. *Vice versa*, reverse COMPARE analysis was done to find factors associated with arsenic trioxide sensitivity. Only correlations with a correlation coefficient of R>0.5 (standard COMPARE) or R<−0.55 (reverse COMPARE) were considered (**Table S1**).

Among the genes identified by this approach were genes from diverse functional groups such as signal transduction (*SYDE1, SFN, PPAP2C, EZR, GPRC5A*), DNA biosynthesis and transcriptional regulation (*UPRT, MED12, SFRS15*), adhesion and cytoskeletal organization (*PDLIM5, PERP, DSG2, SDC1*) and others (*ID1, ILKAP, HMIX2, UBA1, ARHGEF6, CYTH1, TXNRD1, CMTM4*).

**Figure 3:**
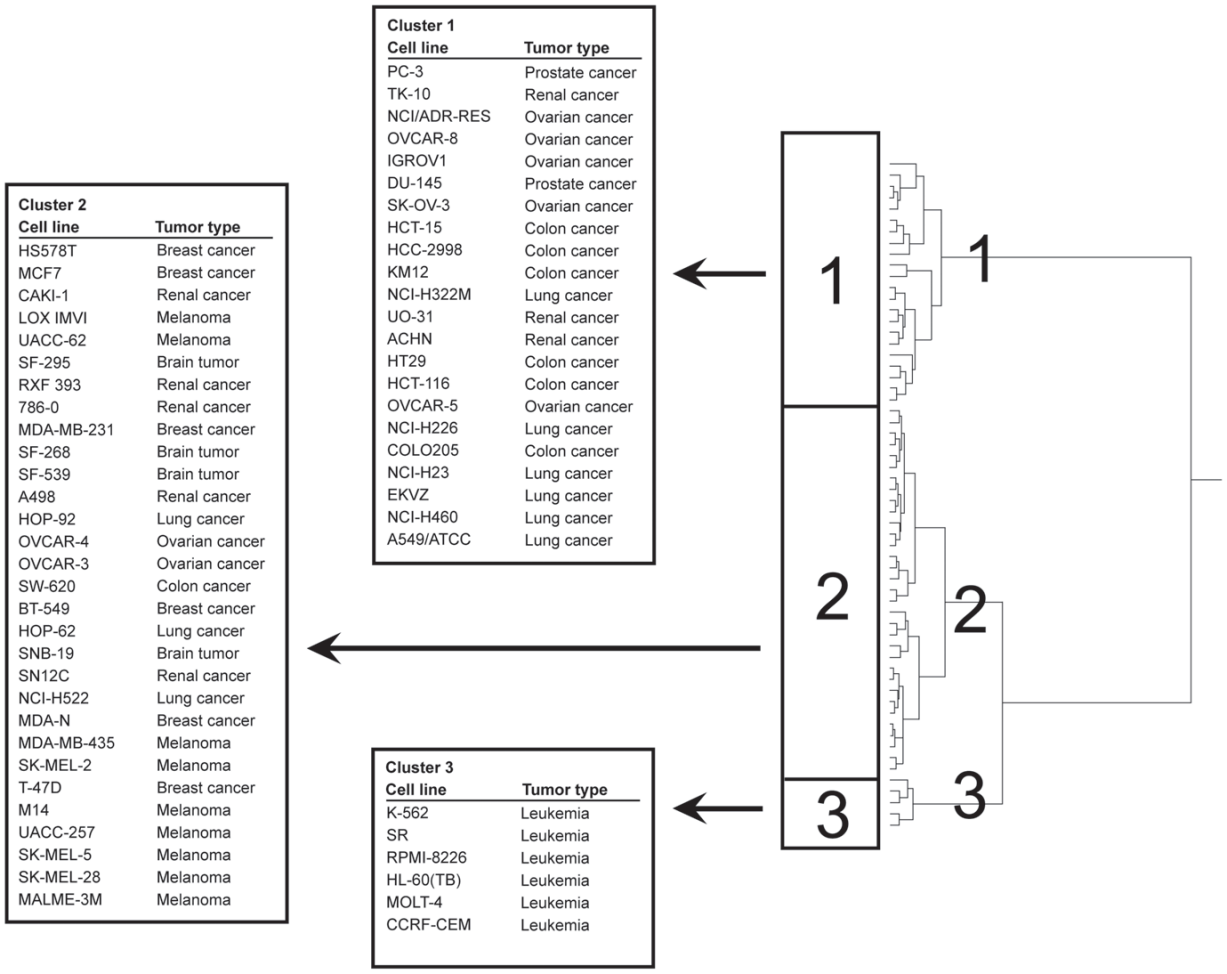
Hierarchical cluster analysis of microarray-based mRNA gene expression obtained by standard and reverse COMPARE analyses. The dendrogram shows the clustering of the NCI-60 cell line panel and indicates the degrees of relatedness between cell lines.

Next, the genes identified by standard and reverse COMPARE analyses were subjected to hierarchical cluster analysis. The dendrogram obtained by this procedure can be divided into three major branches (**Figure 3**). The distribution of cell lines being sensitive or resistant to formulated arsenic trioxide was significantly different between the branches of the dendrograms. The sensitive/resistant ratio in cluster 1 was 2∶20, 22∶10 in cluster 2 and 6∶0 in cluster 3. The distribution of cell lines among the dendrogram predicted resistance to formulated arsenic trioxide with significance (P = 3.2×10^−6^; Chic^2^-test; **Table 2**). A similar relationship was found for free arsenic trioxide (P = 4.5×10–6; Chic^2^-test; **Table 2**).

**Table 2:** Separation of clusters of the NCI cell line panel obtained by the hierarchical cluster analysis shown in **Figure 3** in comparison to drug sensitivity.

		Partition^a^	Cluster 1	Cluster 2	Cluster 3	Chi^2^ Test
formulated arsenic trioxide	sensitive	≤−5.346	2	22	6	
	resistant	>−5.346	20	10	0	3.32635×10^−6^
free arsenic trioxide	sensitive	≤−5.467	2	21	6	
	resistant	>−5.467	20	10	0	4.50505×10^−6^

a The median log_10_IC_50_ value (M) for each drug was used as a cut-off to separate tumor cell lines as being "sensitive" or "resistant".

### Signaling Pathway Profiling

**Figure 4:**
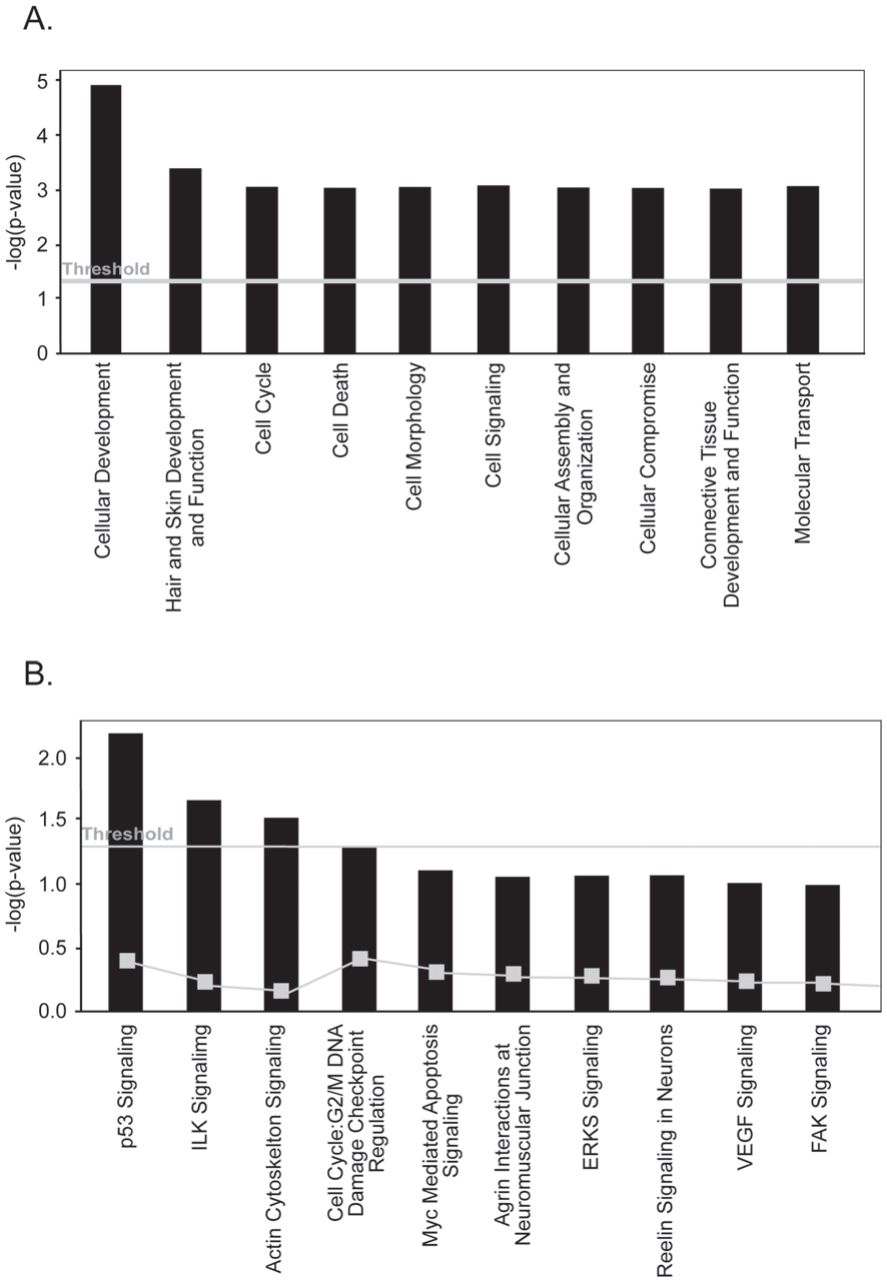
Identification of signaling pathways and interaction of gene products associated with cellular response of cancer cells towards formulated arsenic trioxide. The genes identified by COMPARE analyses (**Table S1**) were subjected to Ingenuity Pathway Analysis Software. (**A**) Top 10 categories of biological functions of the candidate genes. (**B**) Top 10 canonical signaling pathways, which the candidate genes were assigned to.

As a next step, we employed a signaling pathway analysis to better understand the biological consequences of arsenic trioxide treatment. The genes identified by microarray and COMPARE analyses were subjected to Ingenuity Pathway Analysis (version 6.5). The genes identified by COMPARE analysis have a function in cellular development, hair and skin development and function, cell cycle, cell death, and cell morphology and others (**Figure 4A**). The top canonical pathways were signaling routes for p53, ILK, and actin cytoskeleton (**Figure 4B**).

### Candidate Gene Approach

In the second part of our investigation, we analyzed whether classical mechanisms of resistance towards established anti-cancer drugs would also affect response of tumor cells towards arsenic trioxide.

#### Role of oxidative stress response, damage, or metabolism for resistance to arsenic trioxide

**Figure 5:**
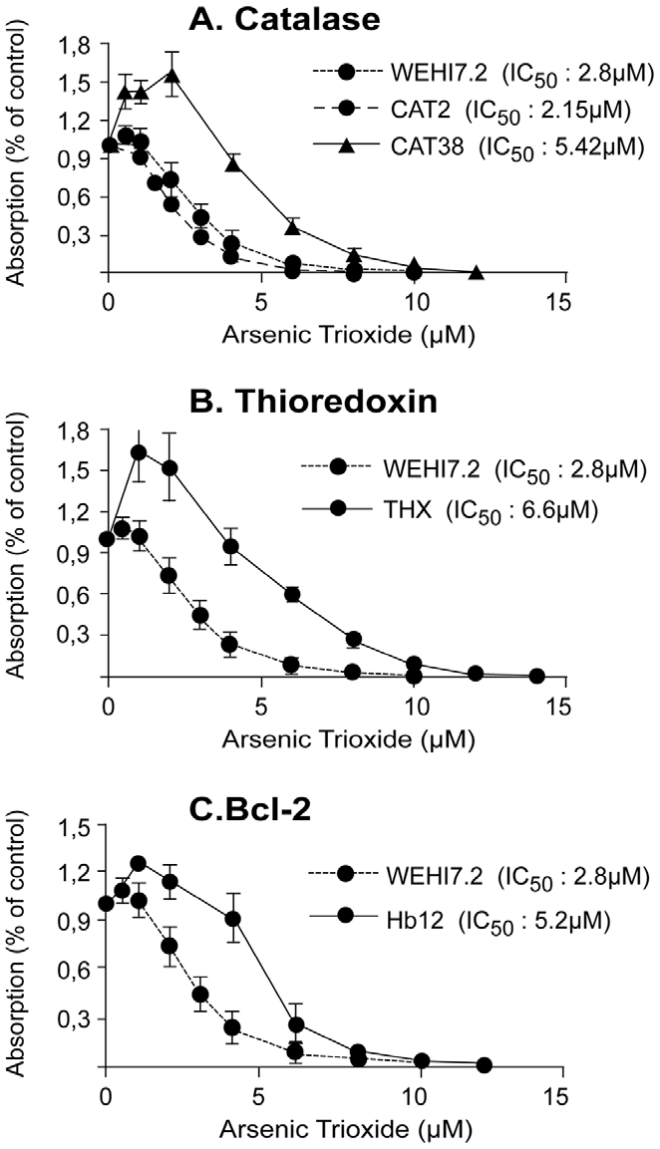
Cytotoxicity of arsenic trioxide on WEHI7.2 cell lines. Cells stably transfected with expression vectors carrying cDNAs for (A) catalase, (B) thioredoxin, or (C) Bcl-2, and with mock control vector. Values represent the mean (± SEM) of three independent experiments.

**Figure 5** shows the arsenic trioxide response of WEHI7.2 mouse thymic lymphoma cells selected for resistance to H_2_O_2_ or stably transfected with catalase, thioredoxin, or bcl-2. The CAT38 clone was 1.94-fold more resistant to arsenic trioxide than the parental WEHI7.2 cells (**Figure 5A**). Thioredoxin-transfected cells were 2.36-fold more resistant to arsenic trioxide than WEHI7.2 cells (**Figure 5B**). WEHI7.2 cells selected for resistance to H_2_O_2_ were not resistant to arsenic trioxide (data not shown). Finally, bcl-2-transfected cells were 1.86-fold more resistant to arsenic trioxide than WEHI7.2 cells (**Figure 5C**).

#### Role of ABC-Transporters for Resistance to Arsenic Trioxide

**Figure 6:**
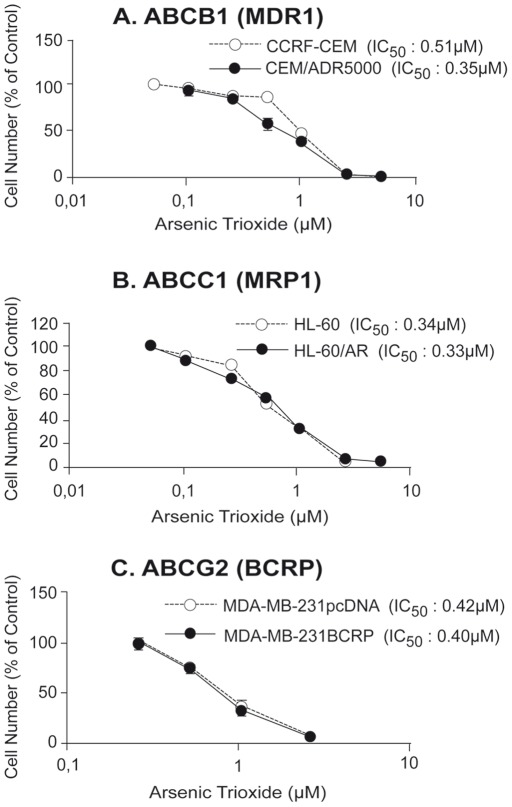
Cytotoxicity of sensitive and multidrug-resistant tumor cells to arsenic trioxide. (A) Sensitive CCRF-CEM and multidrug-resistant *ABCB1* (*MDR1*)*-*overexpressing CEM/ADR5000 cells; (B) sensitive HL60 and multidrug-resistant *ABCC1* (*MRP1*)*-*overexpressing HL60/AR cells; and (C) sensitive MDA-MB-231-pcDNA and multidrug-resistant *ABCG2* (*BCRP*)*-*transduced MDA-MB-231-BCRP cells. Values represent the mean (± SEM) of three independent experiments.

As multidrug resistance (MDR) and MDR-conferring drug transporters of the ABC transporter family are a major cause of failure to many established anti-cancer drugs, we addressed the question, whether cellular response to arsenic trioxide treatment may also be affected by ABC transporters. The role of three ABC transporters has been exemplarily validated using cell lines that selectively overexpress either the *ABCB1* (*MDR1*), *ABCC1* (*MRP1*), or the *ABCG2* (*BCRP*) gene. Based on the IC_50_ values calculated from the dose response curves shown in **Figure 6A**, *ABCB1* (*MDR1*)*-*overexpressing CEM/ADR5000 cells were slightly more sensitive to arsenic trioxide as compared to parental CCRF-CEM cells (degree of increased sensitivity: 0.69). *ABCC1*(*MRP1*)-overexpressing HL60/AR cells and *ABCG2* (*BCRP*)-overexpressing MDA-MB-231-BCRP cells were not more resistance to arsenic trioxide than their drug-sensitive counterparts (**Figure 6B and C**).

## Discussion

### Gene-hunting Approach

In the present investigation, we analyzed molecular determinants of sensitivity and resistance of cancer tumor cell lines towards arsenic trioxide. In general, there are two ways to reach this goal: (1) gene-hunting and (2) candidate gene approaches. Applying the first approach, we correlated the IC_50_ values for arsenic trioxide of 60 tumor cell lines with the microarray-based transcriptome-wide mRNA expression levels of this cell line panel [39] by COMPARE analysis. This approach has been successfully used to unravel the mode of action of novel compounds [43]. Cluster and COMPARE analyses are also useful for comparing gene expression profiles with IC_50_ values for investigational drugs to identify candidate genes for drug resistance [44] and to identify prognostic expression profiles in clinical oncology [45].

We identified genes from diverse functional groups, which were tightly associated with the response of tumor cells to arsenic trioxide, such as genes belonging to p53 signaling and others, most of which have not been associated with cellular response to arsenic trioxide. Interestingly, the oxidative stress response and DNA repair (*TXNRD1* and *UBA1*) appeared in the COMPARE analysis, which speaks DNA damage as mode of action of arsenic trioxide. The gene-hunting approach applied by us delivered several novel candidate genes that may regulate the response of cancer cells to arsenic trioxide. These results merit further investigation to prove the contribution of these genes to arsenic trioxide resistance.

The microarray technology has also been applied by other investigators to analyze genes potentially relevant for cellular response towards arsenic trioxide [46]–[49]. In these studies, the gene expression between untreated and arsenic trioxide-treated cell lines has been compared to identify genes up- or down-regulated upon drug challenge. This approach delivers genes as a response to cytotoxic stress and is different from our approach. In the present investigation, we correlated the basal gene expression of untreated cells in a panel of 60 cell lines with their IC_50_ values to arsenic trioxide. These two experimental settings refer to two different types of drug resistance. The first approach may unravel genes conferring resistance after drug treatment. This type is called acquired or secondary resistance. In our approach, we identify genes involved in the initial responsiveness of tumor cells to drug treatment. This type is known as inherent or primary resistance. Both types of drug resistance can clinically be observed. As an example, small cell lung cancer frequently responds well to chemotherapy at the beginning of a therapy, but gradually develops resistance during subsequent treatment courses (acquired or secondary resistance). Non-small cell lung cancers do not respond well to chemotherapy even at the beginning of a treatment (inherent or primary resistance). This implies that those tumors express drug resistance mechanisms prior to drug treatment.

It is interesting to note that the microarray analysis in the current study identified genes from functional groups similar to those that previous studies identified as associated with cellular response to arsenic trioxide. These include cell cycle-regulating genes [Bibr pone.0035584-Ahn1] [Bibr pone.0035584-Zheng1] [Bibr pone.0035584-Zhao1] [46−49], transcription factors and cofactors [47], [49], signal transducers [46], [50], DNA repair genes [46], [49] and apoptosis-regulating genes [47]. This indicates that these cellular functions may be of importance for resistance to arsenic trioxide. The appearance of these genes was a clue for the involvement of reactive oxygen species (see above), which was indeed validated by our subsequent experiments. Liu et al. [51] also identified oxidative stress response genes and proteins related to the NRF2 pathway in the NCI-60 cell line panel as possible determinants of response to arsenic trioxide. In our approach, we analyzed not the entire set of significantly correlating genes as Liu and colleagues did, but only the genes with the highest COMPARE ranks. Here, genes related to p53 signaling, cell cycle arrest, DNA repair, and apoptosis provide clues on reactive oxygen species as underlying mechanism. Therefore, the report of Liu et al. and the present investigation do nicely complement each other and strengthen the hypothesis of oxidative stress response as important mechanism for arsenic trioxide’s response in cancer cells.

Additional functional groups of genes, which did not appear in the present investigation, were proteasome degradation, RNA processing calcium signaling, the IFN pathway and protein synthesis [47], [50]. Other arsenic trioxide effects include impairment of the genomic differentiation program in human macrophages [52] and alterations in the expression of multiple micro-RNAs. A more detailed analysis is required to determine the relative importance of the multiple effects in the observed drug response.

### Candidate Gene Approach

As a second approach, we analyzed whether several classical drug resistance mechanisms may also play a role for the resistance towards arsenic trioxide. These classical mechanisms did not appear in our COMPARE analyses, although their mRNA expression values were also included into the analysis. This indicates that the above genes identified by COMPARE might be more relevant for response of tumor cells towards arsenic trioxide. Nevertheless, the role of those classical drug resistance mechanisms is worth investigating, because of their generally accepted role for drug resistance to anti-cancer agents.

It has been demonstrated that arsenic trioxide generates ROS (preferentially H_2_O_2_ but also O_2_^•-^ [22]–[24], [53]) and that the cytotoxic activity of arsenic trioxide is reduced by N-acetylcysteine [27], [54]–[56] and enhanced by buthionine sulfoximine [54], [57], [58]. These results imply that oxidative stress induced by arsenic trioxide is important for cytotoxicity. Therefore, it is surprising that contradictory results have been reported for ROS-detoxifying enzymes. Either increased, decreased, or unchanged enzymatic activities upon cellular challenge with arsenic trioxide have been observed for glutathione S-transferase-pi [25], [26], [58], glutathione peroxidase [25], [59]–[61], glutathione reductase [25], [60], catalase [27], [55], [60], [62], [63], superoxide dismutases [25], [27], [56], [61] and thioredoxin reductase [64]. To clarify the role of ROS-detoxifying enzymes, it may not be sufficient to measure enzymatic activities. Therefore, we have used cell lines transfected with cDNAs for catalase or thioredoxin and treated them with arsenic trioxide. We found that transfection of catalase cDNA or thioredoxin cDNA conferred resistance towards arsenic trioxide. In addition to the glutathione redox system, the thioredoxin system represents another major antioxidant system maintaining the intracellular redox state. Thioredoxin scavenges ROS, regulates antioxidant enzymes, and inhibits proapoptotic proteins [65]. Oxidized thioredoxin is reduced by thioredoxin reductase, which is relevant for arsenic trioxide’s activity as shown in the present investigation.

It is unclear from the literature, whether arsenic trioxide induces apoptosis and whether Bcl-2 is protective. This is further complicated by the conflicting data indicating that arsenic trioxide can up- and down-regulate the apoptosis-regulating *bcl-2*, *bcl-x_L_*, and *bax* genes depending on the model system [18], [66]–[68]. Therefore, we attempted to clarify the role of the anti-apoptotic *bcl-2* gene by treating *bcl-2* transfected cells with arsenic trioxide. As expected, we observed that *bcl-2* mediated resistance to this compound providing evidence for the importance of the mitochondrial pathway of apoptosis for arsenic trioxide’s cytotoxicity towards cancer cells.

### ABC Transporters and Multidrug Resistance

Multidrug resistance (MDR) is based on numerous mechanisms, one of which is the influence of ATP-binding cassette (ABC) transporters. They are involved in the active transport of phospholipids, ions, peptides, steroids, polysaccharides, amino acids, bile acids, pharmaceutical drugs and other xenobiotic compounds [16]. ABCB1 (P-glycoprotein, P-gp, MDR1), ABCC1-C6 (MRP1-6) and ABCG2 (BCRP) confer resistance to cytostatic drugs of tumors and contribute to the failure of tumor [17]. It is still unclear, to which extent human ABC transporters contribute to arsenic trioxide-related drug resistance phenomena.

While some authors found no cross-resistance or even collateral sensitivity of cell lines overexpressing P-glycoprotein (*MDR1, ABCB1*) [18], [69]–[71] or *MRP1 (ABCC1)* [18], [20], [21], [72], [73], others claim a role of these ABC transporters in arsenic trioxide resistance [19], [74]. This discussion, *i.e.* whether arsenic trioxide leads to an induction or repression of these two drug transporters, is controversial [66], [70], [75]–[80]. The role of BCRP (ABCG2), another important multidrug resistance-conferring ABC-transporter has not been addressed as yet. For this reason, we have analyzed multidrug-resistant CBM/ADR5000 cells which specifically overexpress P-glycoprotein, but none of the other ABC transporters [17], [30]. These cells were slightly more sensitive to arsenic trioxide, indicating that P-glycoprotein does not play a major role for resistance to this drug. Furthermore, we have analyzed HL60/AR cells, which have been reported to overexpress MRP1 [81]. In a previous investigation we found that other transporters [82] are also overexpressed in this cell line. Since this cell line did not reveal cross-resistance to arsenic trioxide, we conclude that these ABC transporters are not relevant for resistance towards this drug. Likewise, MDA-MB-231/BCRP cells transfected with a cDNA for *BCRP* were not cross-resistant to arsenic trioxide. In summary, our data do not support that the ABC-transporters P-gp, MRP1 and BCRP considerably contribute to resistance to arsenic trioxide. This indicates that clinically refractory tumors overexpressing these ABC transporters might still be responsive to arsenic trioxide.

### Conclusions

In the present investigation, we analyzed molecular determinants of sensitivity and resistance of cancer tumor cell lines to arsenic trioxide. By the gene-hunting approach, we identified genes, which were not yet known to be linked to responsiveness of cancer cells towards arsenic trioxide-. These genes need to be investigated in more detail in future studies. By the candidate gene approach, we analyzed the role of several classical drug resistance mechanisms for the resistance towards arsenic trioxide-apoptotic *bcl-2* gene as well as the thioredoxin reductase gene. ABC transporters were not responsible for resistance to arsenic trioxide (MRP1, BCRP).

Our approach clearly revealed that response of tumor cells towards arsenic trioxide is multi-factorial. At least some of the functional groups of genes are also implicated in clinical responsiveness of tumors towards chemotherapy. Whether the genes identified in the present study also determine clinical responsiveness to arsenic trioxide merits further investigation.

## Supporting Information

Table S1 **Genes determining sensitivity or resistance towards formulated arsenic trioxide in the NCI cell line panel as identified by microarray mRNA expression profiling and COMPARE analysis (see Supporting Information).**
(DOC)
