# Supplementary material for: Factors Determining Sensitivity and Resistance of Tumor Cells to Arsenic Trioxide
Source: PLoS One. 2012 May 10;7(5):e35584. doi: 10.1371/journal.pone.0035584 (PMC3349672; doi:10.1371/journal.pone.0035584)
Supplement: Table S1 — Genes determining sensitivity or resistance towards formulated arsenic trioxide in the NCI cell line panel as identified by microarray mRNA expression profiling and COMPARE analysis (see Supporting Information). (DOC) [file pone.0035584.s001.doc]

**Table S1:** Genes determining sensitivity or resistance towards formulated arsenic trioxide in the NCI cell line panel as identified by microarray mRNA expression profiling and COMPARE analysis.

| **COMPARE** | **Gene** | **GeneBank** | **Pattern** | **Name** | **Function** |
| --- | --- | --- | --- | --- | --- |
| **Coefficient** | **Symbol** | **Acc** | **ID** |  |  |
| **Standard COMPARE:** | |  |  |  |  |
| 0.608 | *UPRT* | N35784 | GC14209 | Uracil phosphoribosyl- | DNA biosynthesis |
|  |  |  |  | Transferase |  |
| 0.591 | *MED12* | NM_005120 | GC183734 | Mediator complex subunit 12 | RNA polymerase II transcription regulation |
| 0.564 | Unknown | AL022398 | GC81812 | Unknown | Unknown |
| 0.557 | *CXorf40B* | AA039366 | GC17149 | Chromosome X open | Unknown |
|  |  |  |  | reading frame 40B |  |
| 0.531 | *SFRS15* | AI041854 | GC154969 | Splicing factor, arginine/serine-rich 15 | Linkage of transcription and pre-mRNA processing |
| 0.529 | Unknown | AI829325 | GC76778 | Unknown | Unknown |
| 0.523 | Unknown | AI077365 | GC58308 | Unknown | Unknown |
| 0.515 | *ILKAP* | AA034514 | GC18631 | Integrin-linked kinase-associated | Serine/threonine phosphatase of the PP2C family |
|  |  |  |  | serine/threonine phosphatase 2C |  |
| 0.507 | *HMOX2* | AI673743 | GC69766 | Heme oxygenase 2 | heme degradation |
| 0.508 | *UBA1* | AA054312 | GC10187 | Ubiquitin-like modifier activating enzyme 1 | Ubiquitin-mediated protein degradation, DNA repair |
| 0.504 | *ARHGEF6* | D25304 | GC177914 | RAC/Cdc42 guanine nucleotide | Signal transduction, Ras-like Rho GTPase |
|  |  |  |  | exchange factor (GEF) 6 |  |
| 0.502 | Unknown | AA805653 | GC50342 | Unknown | Unknown |
| **Reverse COMPARE:** | |  |  |  |  |
| -0.6 | *SYDE1* | AA031652 | GC18470 | Synapse defective 1, Rho GTPase, | Signal transduction; |
|  |  |  |  | homolog 1 (*C. elegans*) | GTPase activator for the Rho-type GTPases |
| -0.577 | Unknown | W74078 | GC16172 | Unknown | Unknown |
| -0.562 | *SFN* | BC000329 | GC171279 | Stratifin | Signal transduction; activator of Akt/mTOR pathway; |
|  |  |  |  |  | TP53-regulated inhibitor of G2/M progression |
| -0.561 | Unknown | AA039610 | GC18882 | Unknown | Unknown |
| -0.555 | Unknown | AA015819 | GC39825 | Unknown | Unknown |
| -0.553 | *PPAP2C* | AF035959 | GC37777 | Phosphatidic acid phosphatase type 2C | Signal transduction; |
|  |  |  |  |  | de novo synthesis of glycerolipids"$C儤 ଀""Integrin-linked kinase-a |
| -0.551 | *CYTH1* | AA041528 | GC18902 | Cytohesin 1 | ARF guanyl-nucleotide exchange factor |
| -0.549 | *PDLIM5* | AA045803 | GC9870 | PDZ and LIM domain 5 | LIM domain zinc finger protein |
|  |  |  |  |  | involved in cytoskeleton organization, cell lineage |
|  |  |  |  |  | specification, organ development, and oncogenesis |
| -0.547 | *PERP* | N95055 | GC15326 |  | Role in cell-cell adhesion by promoting desmosome |
|  |  |  |  |  | assembly; effector in the TP53-dependent apoptosis |
| -0.518 | Unknown | X57348 | GC100341 | Unknown | Unknown |
| -0.516 | *DSG2* | BF031829 | GC174509 | Desmoglein 2 | Component of intercellular desmosome junctions; |
|  |  |  |  |  | cell-cell junction; calcium-binding transmembrane |
|  |  |  |  |  | glycoprotein component of desmosomes |
| -0.515 | Unknown | AL049699 | GC34801 | Unknown | Unknown |
| -0.514 | *EZR* | AA779712 | GC50097 | Ezrin | Protein-tyrosine kinase substrate in microvilli. |
| -0.513 | Unknown | X57348 | GC192177 | Unknown | Unknown |
| -0.51 | *ID1* | X77956 | GC27014 | Inhibitor of DNA binding 1 | Basic helix-loop-helix (HLH) transcription factor |
|  |  |  |  |  | Role in cell growth, senescence, and differentiation |
| -0.508 | Unknown | R79560 | GC12455 | Unknown | Unknown |
| -0.508 | Unknown | X57348 | GC100342 | Unknown | Unknown |
| -0.507 | *TXNRD1* | AA055408 | GC10380 | Thioredoxin reductase 1 | Role in selenium metabolism and protection |
|  |  |  |  |  | against oxidative stress |
| -0.506 | *CMTM4* | W46185 | GC15744 | CKLF-like MARVEL transmembrane | Member of the chemokine-like factor superfamily |
|  |  |  |  | domain containing 4 |  |
| -0.503 | Unknown | X57348 | GC192176 | Unknown | Unknown |
| -0.501 | *SDC1* | R00830 | GC12065 | Syndecan 1 | Transmembrane (type I) heparan sulfate proteoglycan |
|  |  |  |  |  | Syndecans mediate cell binding, cell signaling, |
|  |  |  |  |  | and cytoskeletal organization |
| -0.5 | *GPRC5A* | NM_003979 | GC182879 | G protein-coupled receptor, family C, | Signal transduction; |
|  |  |  |  | group 5, member A | Member of type 3 G protein-coupling receptor family |
|  |  |  |  |  | Role in embryonic development and |
|  |  |  |  |  | epithelial cell differentiation |
